# Textural analysis and lung function study: Predicting lung fitness for radiotherapy from a CT scan

**DOI:** 10.1259/bjro.20180001

**Published:** 2019-04-29

**Authors:** Iain Phillips, Veni Ezhil, Mohammad Hussein, Christopher South, Andrew Nisbet, Sheaka Alobaidli, Vineet Prakash, Mazhar Ajaz, Helen Wang, Philip Evans

**Affiliations:** 1 Royal Surrey County Hospital, Guildford, UK; 2 National Physical Laboratory, Teddington, London, UK; 3 Kuwait Cancer Centre, Kuwait, Kuwait; 4 University of Surrey & Royal Surrey County Hospital, Guildford, UK; 5 University of Surrey, Guildford, UK

## Abstract

**Objective:**

This study tested the hypothesis that shows advanced image analysis can differentiate fit and unfit patients for radical radiotherapy from standard radiotherapy planning imaging, when compared to formal lung function tests, FEV1 (forced expiratory volume in 1 s) and TLCO (transfer factor of carbon monoxide).

**Methods:**

An apical region of interest (ROI) of lung parenchyma was extracted from a standard radiotherapy planning CT scan. Software using a grey level co-occurrence matrix (GLCM) assigned an entropy score to each voxel, based on its similarity to the voxels around it.

**Results:**

Density and entropy scores were compared between a cohort of 29 fit patients (defined as FEV1 and TLCO above 50 % predicted value) and 32 unfit patients (FEV1 or TLCO below 50% predicted). Mean and median density and median entropy were significantly different between fit and unfit patients (*p* = 0.005, 0.0008 and 0.0418 respectively; two-sided Mann–Whitney test).

**Conclusion:**

Density and entropy assessment can differentiate between fit and unfit patients for radical radiotherapy, using standard CT imaging.

**Advances in knowledge:**

This study shows that a novel assessment can generate further data from standard CT imaging. These data could be combined with existing studies to form a multiorgan patient fitness assessment from a single CT scan.

## Introduction

CT scans are ubiquitous in the management of patients with lung cancer. This imaging presents a pool of potential additional information that could be extracted and interpreted. Extracting quantitative features from images and analysing them is termed radiomics.^1^ Textural analysis (TA) is a subtype of radiomics, based on mathematical derivations rather than prior clinical concepts.^2^ The aim of the TEAL (Texture Analysis and Lung function) study is to see whether extra clinical data can be extracted from standard clinical imaging from patients with lung cancer.

TA uses a range of mathematically calculated features to describe an image or region of interest (ROI) within an image. Although different textural features have been generated from a wide range of sources, they can be broadly divided into three categories: first-order (least complex), second-order and higher-order (most complex). First-order features are often calculated as a single value describing the distribution of pixel values. Second-order features describe the relationship between two points, such as two adjacent pixels or voxels within the same image and as such describe more complex relationships. This study uses first-order (density) and second-order (entropy) TA methods.

The vast majority of TA used to assess images from patients with lung cancer has been used to assess malignant tissue,^3,4^ however, CT data can be used to assess normal tissue. For example, cardiac CT may be able to detect global left ventricular function and was able to assess cardiac wall motion abnormalities with a sensitivity of 90% and specificity of 97%.^5^ Previous studies in this area have predicted that forced expiratory volume in 1 s (FEV1) correlated with mean lung density, although this was limited by the need to analyse the whole lung.^6^ Other studies have been able to show that the transfer factor for the lung for carbon monoxide (TLCO) and FEV1 were able to correlate with volume of emphysema.^7–11^ However, no CT studies have been used to assess fitness for radiotherapy.

The aim of this study was to determine whether more advanced analysis of a radiotherapy planning CT could establish lung function and subsequently fitness for radical radiotherapy. By gaining greater information more from existing investigations, it would allow quicker clinical decision making.

### Assessing lung function

Lung function tests can be used to diagnose underlying lung conditions, such as chronic obstructive pulmonary disease (COPD). Both lung cancer and COPD are commonly associated with smoking exposure.

Lung function tests measure many different parameters. FEV1 can be used in combination with clinical history to diagnose COPD and used alone to grade COPD severity using the GOLD criteria.^12^ A predicted value is generated, which is standardised by the patient’s height, age and gender. The value obtained by an individual is expressed as a percentage of the predicted value, the lower the value the more severe the COPD. Although FEV1 is a useful marker of lung function, it should be combined with clinical judgement and other markers of lung function.^13^

TLCO measures the ability of the lung to perform efficient gas exchange between the alveoli and red blood cells. In a stable patient without an acute medical condition, measured TLCO can be used as a marker of lung fitness/function, when expressed as a percentage of the predicted value.

In clinical assessment of lung function, the value generated by the patient is compared to an ideal/“normal value for a patient of that age, height and gender. For example, an FEV1 of 50% of the predicted value means the patient is able to expel only half of the volume of air in 1 s, when compared to the expected value.

All patients having radical radiotherapy have their respiratory function assessed by full lung function tests before treatment. However, the majority of data available correlating lung function with radical treatment outcome have been identified in surgical patients rather than radical radiotherapy patients. Guidelines suggest that lower TLCO and FEV1 correlate with higher post-operative mortality.^14^ However, the guidelines by Brunelli et al state that there is little evidence at what level to set a threshold using lung function tests, to decide who is fit for radical radiotherapy. It is likely that radiation oncologists would have a minimum threshold for lung function for fractionated radical radiotherapy, whereas there is no minimum threshold for stereotactic ablative body radiotherapy (SABR).

The fitness assessment for radical radiotherapy depends on a range of factors. These include: pre-treatment lung function, size of the irradiated volume, radiotherapy technique, prescribed dose to the tumour, whether concurrent chemotherapy is given and markers of likely lung toxicity such as V20 (volume of normal lung receiving 20 Grey or more).^15^ However, baseline lung function is important in deciding whether a lung tumour can be included within a radical radiotherapy treatment volume, without significant long-term side effects for the patient.

In this study, we test the hypothesis that the use of a novel assessment of the appearance of an apical segment of lung, from a standard radiotherapy planning CT, it is possible to differentiate between patients who would be fit or unfit for radical radiotherapy.

## methods and Materials

### Patient selection

Local ethical approval was gained for this retrospective study. Sequential patients who had a four-dimensional CT radiotherapy planning scan with i.v. contrast and available lung function tests at a single institution were screened for the study. 29 fit and 32 unfit patients were included. Patients were pseudoanonymised so that their identities were not known by the research team. All patients had the CT scan with the aim of having radical radiotherapy to a tumour in the lung, including both fractionated radical radiotherapy and SABR. By including SABR patients, this ensured patients with poor lung function were included in the study. A fit patient was defined as TLCO and FEV1 of 50% predicted value or greater. Unfit patients had either TLCO or FEV1 below 50% predicted value. These definitions were pragmatically decided upon as definitive thresholds are not available, however, were felt to be clinically relevant as patients with a TLCO and FEV1 >50% would be likely to receive radical radiotherapy, assuming an appropriate level of patient fitness and that the treatment volume was encompassable in a radical radiotherapy volume. As the percentage predicted TLCO and FEV1 were standardised individually for gender, height and age, it was felt it was not necessary to collect these data separately. The data analysis in this study was divided into two stages, first the cohort was divided using clinical criteria into fit and unfit patients, then image analysis was performed on the two groups.

Patients were excluded if they had previous lung surgery or previous radiotherapy, or did not have both TLCO and FEV1 percentage predicted values available.

### Data extraction

All patients had undergone a four-dimensional axial CT scan using a breathing monitoring system (RPM) with 2.5 mm slice thickness on a General Electric Lightspeed Ultra CT scanner (GE, Boston, MA). The entire thorax was imaged and the scan was divided into 10 breathing phases. From this scan an average intensity projection (AVIP) scan was generated. The AVIP image contained all of the planning data and radiotherapy structures and minimal movement would be expected in the apex, so the AVIP was chosen pragmatically for these reasons. Identical ROIs were generated on each CT scan using the Eclipse treatment planning system (Varian Medical Systems, Palo Alto, CA). A volume of interest (VOI) measuring 4 cm radially by 2.5 cm was generated in the apex of the lung contralateral to the tumour. The VOI did not overlap with the treated planning target volume in any patient, so lung parenchyma in this study was felt to be unaffected by tumour. A 4 cm circle was used to draw the structure on 10 consecutive 2.5 mm slices, ensuring that chest wall and rib was excluded superiorly and circumferentially, as well as the aortic arch being excluded inferiorly (see Supplementary Material 1). An apical segment of lung was chosen because centrolobular emphysema is more common in the lung apex. Therefore, differences between those with a greater degree of emphysema were more likely to be seen in the upper lobe.

Supplemental Material

The CT scan was then exported and anonymised within computational environment for radiotherapy research.^16^ The cylindrical ROI was then extracted and analysed using software developed in house.

The data were quantised using the range of grey levels only. The grey levels represented density. The grey levels were quantised into 16 levels, with each 5 × 5 mm voxel being compared in turn to surrounding voxels using a grey level co-occurrence matrix,^17^ using software developed in Matlab 2015.^18^ Previous studies have used multiple slices of a CT scan as a technique of data extraction.^19,20^ A grey level co-occurrence matrix identifies similarities or differences in the grey levels in the 13 directions containing the 26 voxels that surround each voxel being analysed. This built up a voxel by voxel entropy map for each ROI and the entropy score for each voxel was then averaged across the ROI for the final analysis. Averaging the value has been used in a study investigating effects of radiotherapy of lung and tumour tissue.^21^ This study used uniform quantisation.^22^ All of the analyses were standardised to pre-defined quantisation levels from VOI analysis of a fit patient, who was a non-smoker. This meant the electron density values of the quantisation levels for all analyses were fixed and identical for the analysis of all ROIs.

This methodology meant each voxel had a density score and an entropy score. By choosing a single textural feature, we reduced the risk of extracting multiple features and potentially overfitting multiple extracted features to our model^4^ This method also gave a visual entropy map, a numerical analysis and the ability to plot histograms as described below.

### Data analysis

A density score based on the original CT scan was generated and plotted on the X axis, entropy score was plotted on the Y axis, the two-dimensional histogram gave a first visual assessment of differences between fit and unfit patients.

The patients were divided into fit and unfit and results were compared for mean, median and mode density and entropy. Correlation co-efficients would be generated for TLCO and FEV1 against mean, median and modal density and entropy. As the distribution of the data was skewed a Mann–Whitney test was used to compare the two cohorts. Examples of this can be seen in Supplementary Material 1. A moment analysis was then completed on the whole cylinder of lung tissue, enabling a comparison of skewness and kurtosis of fit and unfit patients, the results of this are seen in Table 2 in Supplementary Material 1. (Table 1). Mean, median and mode density and entropy scores were generated for the whole volume.

**Table 1.** *p-*Values of Mann–Whitney test when comparing fit *vs* unfit patients

		Density		Entropy	
		Mean	Median	Mean	Median
Whole volume	Fit	252.05	224.69	2.51	2.43
	Unfit	193.53	166.28	1.97	1.78
	*p*-value	**0.004488**	**0.000792**	0.0736	**0.0418**

Comparison of mean and median density, as well as median entropy between fit and unfit patients are statistically significant.

## Results

Mean FEV1 in the fit group was 85% predicted (range 50–113%) and 53% predicted (range 27–110%) in the unfit group. Mean TLCO in the fit group was 74% predicted (range 52–99%) and 43% predicted (range 29–71%) in the unfit group. Correlations between a single marker of lung function (either TLCO or FEV1) and a single result from image analysis were low. They have been included in Supplementary Material 1. Table 1 illustrates the analysis for the comparison of a threshold of FEV1 50% and TLCO 50% (fit patients) against cohort where either value is below 50% (unfit patients). Alternative thresholds were tested and can be found in Supplementary Material 1. A subregion analysis was decided upon as there appeared to be a difference between the number of data points in the low density low entropy subregion, when fit and unfit patients were compared. The analysis also included some preliminary first order features, including skewness and kurtosis. Using a Mann–Whitney test these were not statistically significant (*p*-value 0.05). The results are in Supplementary Material 1.

## Discussion

This study suggests there are density and entropy differences are detectable when comparing patients who are fit for radical radiotherapy with adequate lung function from patients with poorer lung function who would not be fit for fractionated radical radiotherapy, as SABR has no minimum threshold. Statistically significant differences were established between mean and median CT density and mean, median and skewness entropy scores between fit and unfit patients when using uniform quantisation. CT density is a good discriminator between fit and unfit patients; however, the addition of entropy assessments such as mean, median and skewness of entropy gives additional information. Providing an entropy score for each voxel means that the data plots and the texture maps can be generated (Supplementary Material 1).

The difference in data distribution between fit and unfit patients can be seen in Figure 1 provided in Supplementary Material 1. Figure 1(a) shows a fit patient, Figure 1(b) shows a plot representing an unfit patient.

This study differs from most texture analysis biomarkers studies as it uses entropy as the single texture measurement, which was combined with density. Rather than pursuing a range of measurements, as seen in other studies, the TEAL study aimed to use texture analysis to quantify differences between images of fit and unfit patients, then identify a biological reason for the differences in the images between the two cohorts. This study also aims to avoid the risk of overfitting of multiple assessments of the same image, which could lead to less accurate conclusions.^4^ Entropy has been previously shown to be a measurement that has helped differentiate between good and poor overall survival in non-small cell lung cancer.^23,24^ As a result we wanted to test this on non-malignant tissue. It aims to quantify differences in lung parenchyma structure, which are sometimes visible, but only a qualitative assessment can be made by eye. The fact that unfit patients had lower density and lower entropy values suggests there is more air and less tissue in the ROI. What is not clear is whether TA of lung parenchyma is picking up early microscopic changes of COPD such as bronchiole tissue destruction and alveolar wall destruction or later macroscopic changes such as bullae formation.^25^ For patients with worse COPD, they will likely have poorer lung function (and a lower FEV1) and be more likely to be categorised into the unfit group. More severe COPD would manifest itself as greater destruction of the elastic lung tissue, which leads to the formation of macroscopic holes in the lung known as bullae. Tissue destruction would lead to lower density lung tissue, potentially explaining why unfit patients have a lower density score. The ROI was positioned in the apex as centrolobular emphysematous changes are more likely to be seen in the lung apex. Centrolobular emphysema is the commonest pattern of emphysema seen in smokers.

In relation to entropy, it is not surprising that a higher entropy score is found in the fit patients compared to the unfit patients. Entropy is a measure of disorder. The higher the entropy, the greater the disorder and the lower the uniformity. A section of healthy peripheral lung tissue would contain a variety of tissues, including small airways, blood vessels and alveoli. As a result, it would be expected that a range of different densities would be seen in the section of analysed lung parenchyma. The interaction of these different tissues means it is likely that a voxel would be different to other voxels around it as it contains different tissue to those around it. Patients with a greater degree of COPD would be more likely to have more lung damage exhibiting itself as worse lung function, which in the CT scan could appear as low density homogeneous areas in the texture map (Supplementary Material 1), meaning the voxels have closer values, *i.e*. would have lower entropy. It may be that future work requires multiple ROIs as emphysema can be localised within certain sections of the lung.

By determining that it is possible to differentiate between fit and unfit patients for radical radiotherapy from an existing CT scan, this has the potential to allow quicker clinical decision making with fewer investigations. . These data need to be reproduced on a diagnostic CT and in a larger data set, but a screening test and apical lung texture could be included as standard as part of formal CT reports. This work also suggests a new paradigm in terms of generating new information from CT data. This could take a number of forms in patients with lung cancer. Recent data have shown muscle attenuation on CT imaging affects outcome, this could be extracted from standard CT data.^26^ Although coronary artery calcium scores and a qualitative assessment of the presence of bullae may be reported, however, there is not a formal structure for this and is not routinely done.

Previous studies have suggested that patients undergoing investigations for potential radical treatment take longer than those for palliative treatment for non-small cell lung cancer as they need additional investigations such as formal lung function or positron emission tomography-CT imaging.^27^ This suggests that patients would benefit from quicker treatment if they can undergo fewer tests or more information can be generated from existing tests. To aid this, it may be helpful for CT imaging to have a minimum data set, as seen in many histopathology reports. A standard proforma for histology of localised breast tumours significantly improved the completeness of histopathological reports.^28,29^ This meant that the data needed to make clinical decisions was more complete and potentially clinicians can make appropriate treatment decisions more quickly.

This study shows that it is possible to differentiate between fit and unfit patients in terms of lung function from a single planning CT scan. If objective markers of cardiac function, muscle assessment and respiratory function could be extracted from standard CT data, then potentially this could be reported as standard, adding to patient data and the ease of availability of this data could lead to quicker clinical decisions, particularly in tumour boards or multidisciplinary team meetings.

The correlation coefficients between a single measurement of lung function (either FEV1 or TLCO) and density or entropy were low suggesting that the relationship between lung function, density and entropy was more complex than a correlation with one marker of lung function.

In this study choosing standardised levels was limited by the availability of CT data (in this case radiotherapy planning CT scans). For this comparison, the levels were standardised to the lungs of a patient who was a non-smoker and "fit", providing levels from lung tissue that was least likely to be abnormal. As long as the quantisation levels were fixed for the analysis of all patients, quantising data by standards from a cohort of patients, may not alter the outcome of the analysis. Defining who is has “normal” lungs is more complicated in this study as all patients were due to receive radical radiotherapy for a tumour in the thorax.

The methodology used in this study aimed to keep the analysis process quick and simple. Integrating advanced analysis of CT images using textural analysis or other radiomic approaches must take account of a radiology department’s work flow.^3^ This limits a lot of analyses. A technique to analyse part of the lung rather than the whole lung is attractive in the interests of processing time, analysis of the whole lung would take several hours compared to less than 10 min for the analysis of the cylinder of lung used in this study. Although less detailed than formal pulmonary function tests, this CT analysis is quick and does not require any expertise to identify the ROI. In this study, the ROIs were drawn by hand, but future work will aim to automate it. The ROI could be identified at the time of scan acquisition, meaning it is ready for interpretation by the reporting clinician the first time they view the scan. It could be simply incorporated into standard radiology work flow. The ROI was positioned in the apex of the lung as this meant creating the ROI was as straightforward as possible. By keeping it as apical as possible it meant it was as reproducible as possible.

This technique was used to differentiate fit and unfit patients for radical radiotherapy, but it could potentially be used to screen patients for lung function assessment. As previously discussed, CT markers of cardiac risk and function have been described and it may be possible to derive function from CT imaging.

The volume of imaging used in planning and treating patients with external beam radiotherapy is increasing. Advanced image analysis could have a range of uses in these patients. The TEAL study has shown that when using the correct quantisation method, functional lung data can potentially be derived from a CT scan. It also generates the hypothesis that more data can be easily and quickly extracted from a standard CT scan, leading to quicker clinical decision-making.
