# Supplementary material for: Textural analysis and lung function study: Predicting lung fitness for radiotherapy from a CT scan
Source: BJR Open. 2019 Apr 29;1(1):20180001. doi: 10.1259/bjro.20180001 (PMC7592404; doi:10.1259/bjro.20180001)
Supplement: Supplemental Material [file bjro.20180001.suppl-01.docx]

**Textural Analysis and Lung Function study: Predicting lung fitness for radiotherapy from a CT scan**

**Supplementary materials**

The aim of the supplementary materials is to give a pictorial example of how the region of interest was generated, give a visual example of the texture maps from fit and unfit patients, show that correlations between density or entropy with lung function are low, show the entropy calculation used within the analysis software and to show the distribution of the data and how that then influenced the use of statistical tests.

**Generating the region of interest**

The apex of the lung was chosen for 2 reasons, firstly because this is reproducible. The top slice of the ROI was the most superior slice of the CT image that could fit a 4cm diameter circle that only included lung tissue, not soft tissue or rib. The second reason was that the apex/upper lobe of the lung is the most likely site of centro-lobular emphysema and so was expected to yield the difference between fit and unfit patients. Analysing the same part of the lung lower down and resampling is more difficult to reproduce between patients and is less likely to yield a difference between fit and unfit patients.


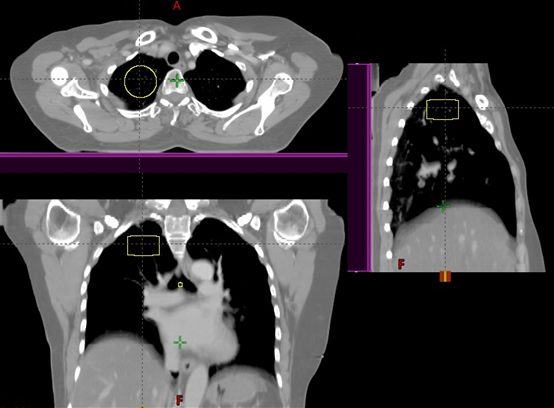


*Figure 1: Yellow outline in right lung illustrates region of interest used for texture analysis in the right lung.*


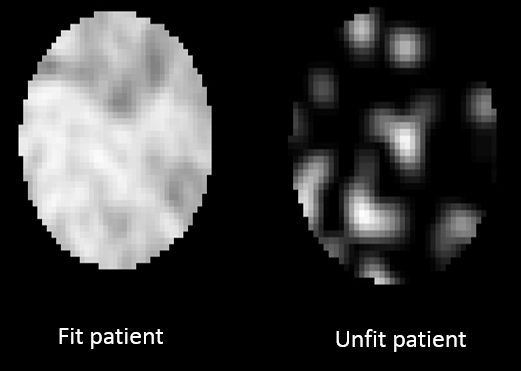


*Figure 2: Visual texture map of a fit patient vs unfit patient. Black illustrates low entropy and white illustrates high entropy*

Correlations between lung function (TLCO or FEV1) versus density and texture markers

|  | TLCO | FEV1 |
| --- | --- | --- |
| Mean density | 0.23 | 0.32 |
| Median density | 0.24 | 0.30 |
| Modal density | 0.20 | 0.28 |
| Mean entropy | 0.21 | 0.33 |
| Median entropy | 0.21 | 0.34 |
| Modal entropy | 0.13 | 0.12 |

*Table 1: Correlation co-efficients between TLCO or FEV1 and the different imaging assessments*

Entropy calculation


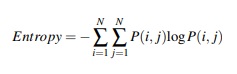


Where P(i,j,) is the probability that a voxel of intensity I has a voxel of intensity j immediately adjacent to it and the sum over i and j is over all intensity values in the image.

Assessment of normal distribution of patients in fit unfit cohort using density


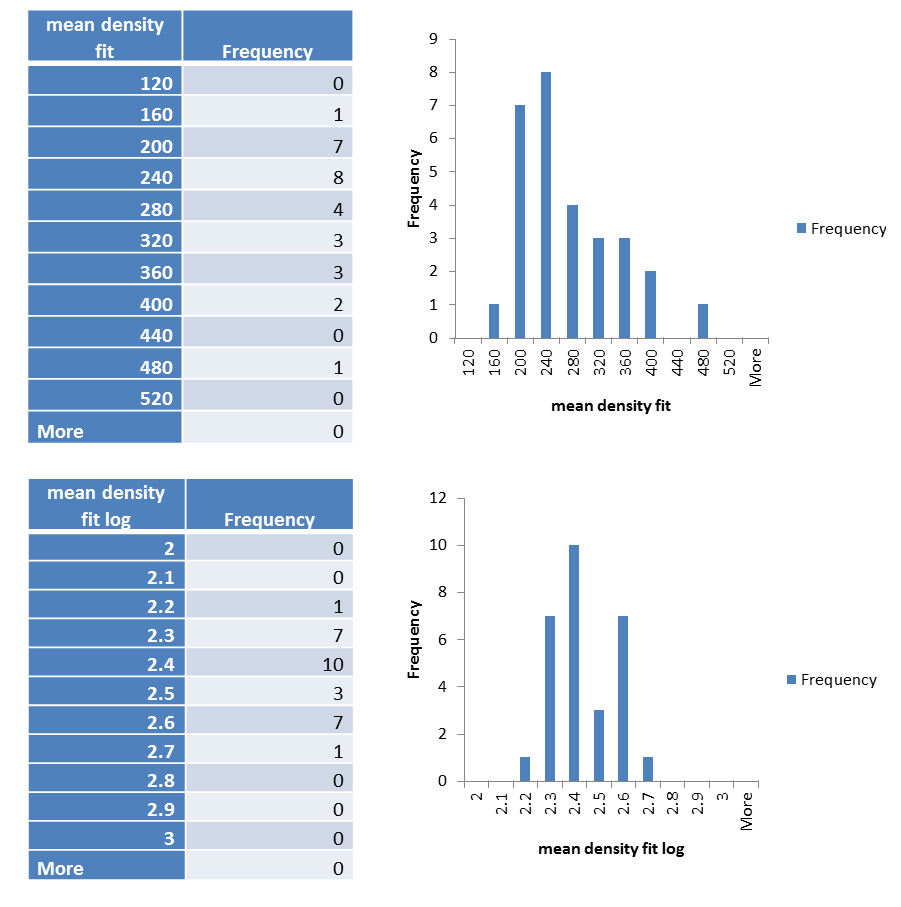


*Figure 3: comparing histograms of mean density for fit patients with log of values for mean density for fit patients. This shows distribution improves with log values. This occurred for mean and median density for fit and unfit patients, however a Shapiro-Wilks test showed the data was not normally distributed.*

Assessment of normal distribution of patients in fit unfit cohort using entropy


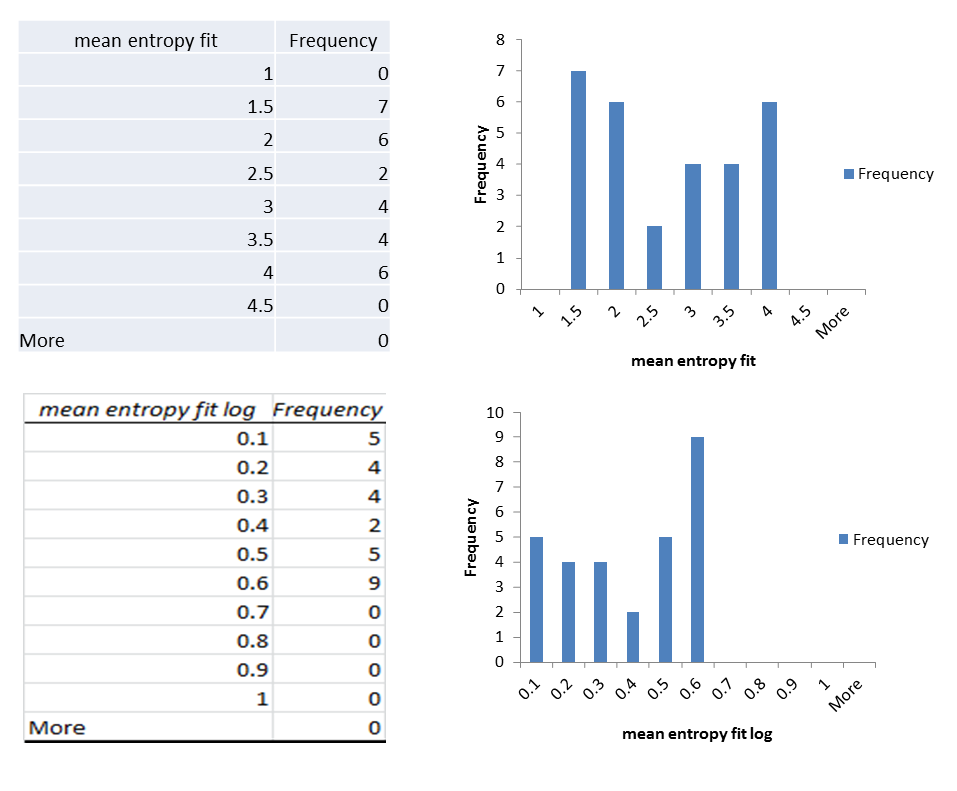


*Figure 4: comparing histograms of mean entropy for fit patients with log of values for mean entropy for fit patients. This shows distribution improved with log values. This occurred for mean and median entropy for fit and unfit patients, however a Shapiro-Wilks test showed the data was not normally distributed.*

**Wilks-Shapiro test**

Although a log10 conversation improved the distribution, as shown if figures 3 and 4 above, it was felt that a formal assessment of distribution was required. In a wilks-shapiro test performed on both mean entropy and mean density, the p value was <0.05, so the null hypothesis could not be rejected. As a result we could not prove that the data in this study was normally distributed. For this reason a Mann-whitney non-parametric test was performed to differentiate between fit and unfit patients.

Other measures of texture for lung function of fit vs unfit patients.

|  | SD |  | Skewness |  | Kurtosis |  |
| --- | --- | --- | --- | --- | --- | --- |
|  | density | entropy | density | entropy | density | entropy |
| fit mean | 101.1 | 1.1 | 2.7 | 0.14 | 13.3 | 2.7 |
| unfit mean | 96.9 | 1.1 | 2.5 | 0.6 | 12 | 2.9 |
| P value of Mann-Whitney test | 0.88 | 0.16 | 0.21 | 0.054 | 0.22 | 0.91 |

*Table 2:* *Results of moment analysis, showing that comparing the different measures of texture analysis are not statistically significantly different (p value 0.05).*

Figure 5: Data from fit patients for skewness, which is then tested using a 2 sided mann-whitney test in table 2.

Measures of texture using different lung function thresholds

|  | FEV1 55% + TLCO 55% predicted | FEV1 50% + TLCO 50% predicted | FEV1 45% + TLCO 45% predicted | FEV1 40% +  TLCO 40% predicted |
| --- | --- | --- | --- | --- |
| Number of fit patients | 24 | 30 | 39 | 46 |
| Number of unfit patients | 38 | 32 | 23 | 16 |
| Mean density p value | 0.03 | 0.0045 | 0.0039 | 0.035 |
| Median density p value | 0.03 | 0.008 | 0.0064 | 0.024 |
| Mean entropy  P value | 0.102 | 0.074 | 0.051 | 0.053 |
| Median entropy p value | 0.073 | 0.042 | 0.03 | 0.03 |

*Table 3: Comparison between different lung function thresholds for fit and unfit patients using a Mann Whitney non parametric test.*
